# Effect of Multi-Walled Carbon Nanotubes and Carbon Fiber Reinforcements on the Mechanical and Tribological Behavior of Hybrid Mg-AZ91D Nanocomposites

**DOI:** 10.3390/ma15176181

**Published:** 2022-09-05

**Authors:** G. U. Raju, Vinod Kumar V. Meti, N. R. Banapurmath, T. M. Yunus Khan, I. G. Siddhalingeshwar, Vishal Vaikunte, Chandramouli Vadlamudi, Sanjay Krishnappa, A. M. Sajjan, Adarsh Patil

**Affiliations:** 1 Department of Mechanical Engineering, K.L.E. Technological University, Hubballi 580031, India; 2 Department of Automation and Robotics, K.L.E. Technological University, Hubballi 580031, India; 3 Centre of Excellence in Material Science, K.L.E. Technological University, Hubballi 580031, India; 4 Department of Mechanical Engineering, College of Engineering, King Khalid University, Asir-Abha 61421, Saudi Arabia; 5 Aerosapien Technologies, Daytona Beach, FL 32114, USA

**Keywords:** magnesium AZ91D composite, multi-walled carbon nanotubes, carbon fibers, tensile strength, impact strength, wear resistance

## Abstract

Magnesium matrix composites are extensively used in automotive and structural applications due to their low density, high strength, and wear-resistant properties. To reach the scope of industry needs, research is carried out regarding enhancing the mechanical and tribological behavior of the magnesium composites by reinforcing the nano-sized reinforcements. In the present work, research has been carried out to enhance the properties of the magnesium AZ91D hybrid composite by reinforcing carbon fibers (CFs) and multi-walled carbon nanotubes (MWCNTs) with varying weight percentages (AZ91D + 0.5% CF’s + 0.5% MWCNT and AZ91D + 0.75% CF’s + 0.75% MWCNT, respectively). The experimental tests were carried out to evaluate the mechanical and tribological behavior of the composites. The test results showed that the addition of CF and MWCNT reinforcements improved the hybrid Mg composite’s hardness, tensile strength, and impact strength compared to the base Mg matrix. The AZ91D + 0.75% CF’s + 0.75% MWCNT hybrid composite showed a 19%, 35%, and 66% increased hardness, tensile strength, and impact strength, respectively, compared to the base Mg AZ91D. The wear test results also showed the improved wear resistance of the Mg composite compared to the base matrix. The enhanced wear resistance of the composite is due to the addition of hard MWCNT and CF reinforcements. The wear rate of the AZ91D + 0.75%CF’s + 0.75% MWCNT composite for a load of 30 N at a sliding distance of 1500 m is lower as compared to the base matrix. The SEM micrographs of the worn surfaces revealed the existence of abrasive wear. The improved mechanical and tribological behavior of the magnesium composite is also due to the homogeneous distribution of the hard reinforcement particles along the grain boundaries.

## 1. Introduction

Magnesium and its alloys are widely used in modern automobile, aerospace, marine, and structural applications, and are predominantly used in high-performance vehicles. The most commonly used magnesium alloys are AZ63, AZ92, and AZ91 for die castings, AZ92 for permanent mold casting, and AZ61 for forging alloy [1]. Due to its excellent mechanical properties, corrosion resistance, and castability properties, AZ91D is the most commonly used magnesium die-cast alloy. Magnesium alloys’ tensile strength, yield strength, and hardness decrease with increasing temperature, while ductility increases [2,3]. Magnesium AZ91D cast alloy can be found in many applications; examples include covers, housings, handheld tools, sporting goods, computer parts, mobile and stationary telephones, household equipment, and automobile components. To improve the existing properties of the mg AZ91D cast alloy, reinforcements are added to form a magnesium composite. Reinforcements are substances that enhance the composite’s hardness, tensile strength, and wear resistance [4,5]. Some reinforcements include SiO2, Y2O3, TiO2, Al2O3, ZrO2, etc. Multi-walled carbon nanotubes (MWCNTs) are a true example of nanotechnology. They have a diameter of fewer than 100 nanometers and can be as thin as 1 or 2 nm. Researchers are exploring their utilization in various applications, such as materials science, electronics, chemical processing, energy management, and other fields [6,7]. Carbon fibers (also known as CF, or graphite fibers) are made primarily of carbon atoms and measure 5 to 10 μm (0.00020–0.00039 in) in diameter. Carbon fibers have several advantages, including high stiffness [8,9,10]. Carbon fibers’ properties have made it very popular in aerospace, civil engineering, military, and motorsports, as well as other competitive sports applications. CF or MWCNTs enhance the hardness and tensile strength of cast composites. Research has proved that the mechanical and tribological properties of the product improved by adding MWCNT reinforcements [11,12].

The performance of magnesium composites depends upon the right combination and composition of reinforcement material with the base magnesium matrix material. Furthermore, reinforcements are added to enhance the mechanical and tribological properties of the magnesium composite. The addition of two reinforcements with varying weight percentages (wt.%) forms hybrid composites [13,14,15]. The hybrid magnesium composites are a new class of metal matrix composites widely used in automotive, marine, aerospace, and structural applications due to their low density, good mechanical properties, better corrosion and wear resistance, and low thermal expansion coefficient compared to conventional metals and alloys [16,17]. Microstructure and morphological changes in carbon nanotube films deposited on different substrates (mica, quartz, and TGX grating) by FESEM and atomic force microscopy was reported previously [18,19]. The evolution of rough-surface geometry and the crystalline structures of aligned TiO_2_ nanotubes for photoelectrochemical water splitting have been discussed [20].

There have been exhaustive literature surveys carried out on the feasibility studies of metal nano-composites using either micro or nano-fillers. However, use of both fillers, highlighting the synergetic effects of these micro and nano fillers in the metal base matrix, has been much less frequently investigated. The novelty of this work involves the synthesis of hybrid metal nanocomposites reinforced with micro fillers of both carbon fiber (CFs) and nano-filler MWCNTs, respectively. These fillers effectively arrest the cracks in the hybrid composites synthesized at both the micro and nano levels, respectively.

Hence, in the present investigation, an attempt has been made to synthesize and evaluate the mechanical and tribological behavior of the hybrid nanocomposite by reinforcing the CFs and MWCNTs.

## 2. Materials and Methods

**Figure 1:**
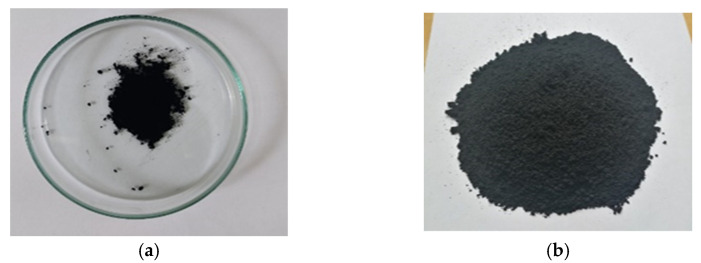
Sample of nano reinforcements; (**a**) carbon fibers and (**b**) MWCNT.

In this research, Mg AZ91D is used as a base matrix material to synthesize the Mg matrix composite. The reinforcing materials used are carbon fibers (CFs) and multi-walled carbon nanotubes (MWCNTs). Carbon fibers are procured from Arrow tex, India, 10 mm in length and 8 µm in diameter. Multi-walled carbon nanotubes were procured from M/s United Nanotech, India. The diameter of the MWCNTs was in the range of 10–30 nm, and their length varied from 1–2 microns. Two reinforcements of MWCNT and CF are used for the synthesis of hybrid nano-composites to address both nano and micro level strengthening. Further, the two reinforcements used adequately enhanced both the mechanical and tribological properties of the Mg AZ91D composites. The varying weight percentages of the two nano and micro fillers were selected based on the improved mechanical properties, after careful optimization of their dosages, respectively. Figure 1a shows the sample of carbon fiber and Figure 1b shows the sample of MWCNT reinforcements. A motorized stir casting, using the bottom pouring technique, is used to prepare the hybrid magnesium nanocomposite.

Carbon fiber and MWCNT, with a weight fraction of 0.5% and 0.75%, respectively, were added to support the synthesis of the AZ91D composites. To improve the wettability of the reinforcements, 1 to 2 wt.% of magnesium was added to the liquid mixture, and mechanical stirring was performed at 400 rpm for 10 min continuously. During the addition of the reinforcement, a constant supply of argon gas was passed into the furnace at 1.8 L/min to avoid contamination from the surrounding environment and avoid the spraying action of nano particulates around the furnace stirring action. All the variables in the control unit were monitored, employing a digital indicator connected to a thermocouple of chrome alum.

**Figure 2:**
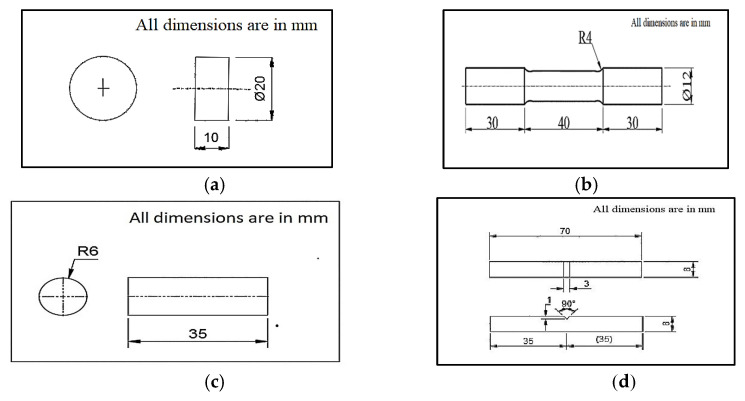
Mechanical characterization as ASTM standards used: (**a**) hardness test specimen dimension [ASTM E92-82]; (**b**) tensile test sample dimension [ASTM E8]; (**c**) wear test specimen dimension [ASTM G99]; (**d**) impact test sample dimension [ASTM E23-16a].

The AZ91D (99.9%) was warmed in a steel crucible and kept in a chip-controlled electric heater under a dormant argon environment. Reinforcement particles were blended with the AZ91D compound to manufacture the required composites using suitable stir casting equipment. The slurry was integrated with the assistance of a hardened steel stirrer at a steady speed of 650 rpm for 15 min at 700 °C, and the liquefied temperature was brought down to 630 °C. It was then quickly warmed to 720 °C and filled into a steel mold measuring 300 mm × 100 mm × 15 mm. Figure 2 shows the mechanical characterization of the slips developed as ASTM standards used.

## 3. Results

### 3.1. SEM Micrographs of AZ91D Hybrid Composites

**Figure 3:**
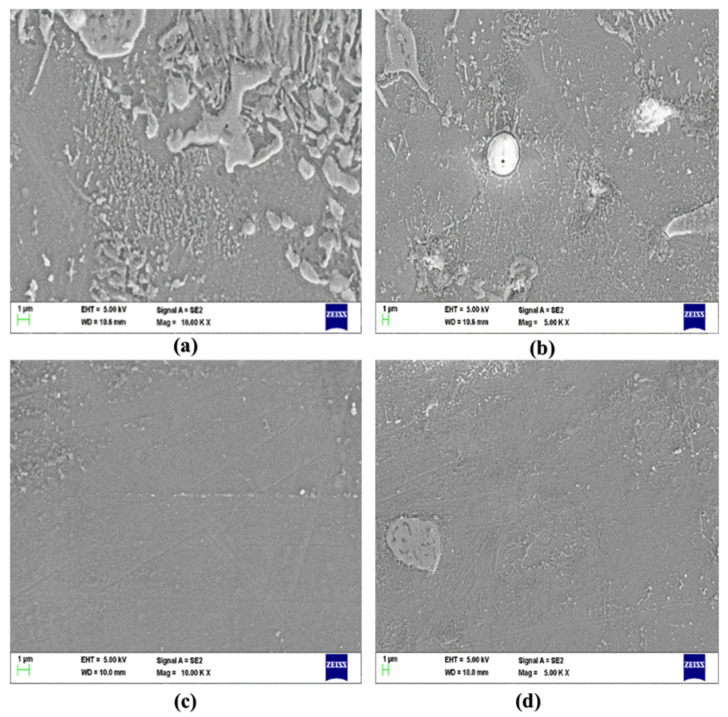
SEM micrographs of AZ91D composite with varying weight percentages of CF and MWCNT reinforcements. (**a**,**b**): AZ91D + 0.5% CF’s + 0.5% MWCNT composite and (**c**,**d**): AZ91D + 0.75% CF’s + 0.75% MWCNT composite.

Figure 3 shows the scanning electron microscope (SEM) micrographs of the magnesium AZ91D composite reinforced with CFs and MWCNTs. The SEM micrographs revealed the presence of CF and MWCNT reinforcements. Figure 3c,d shows a homogeneous distribution of the Mg AZ91D composite having 0.75 wt.% CF and 0.75 wt.% MWCNT reinforcements compared to the Mg AZ91D composite, having 0.5 wt.% CF and 0.5 wt.% MWCNT, as shown in Figure 3a,b. The motorized stir casting technique helped to distribute the reinforcements uniformly along the grain boundaries of the Mg AZ91D composite. The SEM micrographs show the significant grain refinement, homogeneous distribution of reinforcements, and the minimal presence of porosity in the AZ91D hybrid composite. It can also be observed from the SEM micrographs that the reinforcement CF and MWCNT are well bonded with the Mg AZ91D matrix material. Interface bonding analysis was ensured using EDS analysis to confirm the presence of CFs and MWCNTs in the reinforced Mg AZ91D alloy.

The formation of precipitates was observed in the Mg composite or at the interfaces of the Mg composite. The presence of the precipitates at the interfaces influences the properties of the hybrid Mg composite. The precipitates formed were hard and brittle in nature, contributing to the improved mechanical and tribological properties of the hybrid Mg composite. The composite having 0.75 wt.% CF and 0.75 wt.% MWCNT reinforcements showed no agglomeration and segregation of the reinforcements, as well as the absence of porosities, slags, and blow holes compared to the composite having 0.5 wt.% CF and 0.5 wt.% MWCNT reinforcements. The presence of reinforcements along the grain boundaries may act as a barrier to the further deformation of the grains. As a result of the restriction in the further growth of the grains, the primary phase would allow the melt to have enough time to form more nuclei. The homogeneous distribution of the CF and MWCNT reinforcements significantly contributes to enhancing the mechanical and tribological properties of the Mg AZ91D composite.

### 3.2. Hardness of AZ91D Hybrid Composites

**Figure 4:**
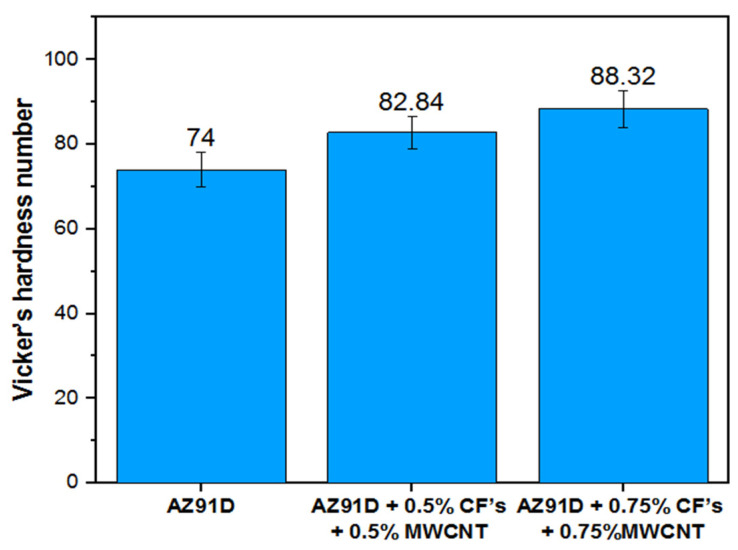
Variation of Vickers hardness number in the Mg AZ91D hybrid composite with varying weight percentages of CF and MWCNT reinforcements.

Figure 4 shows the variations in the Vickers hardness number of the Mg AZ91D hybrid composite reinforced with CFs and MWCNTs. It has been observed from the figure that the addition of CF and MWCNT reinforcements improves the hardness of the Mg hybrid composite compared to the Mg AZ91D. The Mg AZ91D composite with 0.75 wt.% CF and 0.75 wt.% MWCNT reinforcements shows a hardness of 6.5% and 19% higher compared to the Mg AZ91D composite with 0.5 wt.% CF and 0.5 wt.% MWCNT, and Mg AZ91D, respectively. With the addition of hard reinforcements, a strong interfacial bond is generated, which acts as a barrier to the dislocation movement within the reinforcements and matrix material of Mg AZ91D. In the presence of a strong interfacial bond, the material exhibits greater resistance to indentation, due to the hardness of the Mg AZ91D hybrid composite, thereby enhancing the hardness of the hybrid composite. The improved hardness of the hybrid Mg AZ91D composite is also due to the uniform distribution of the CF and MWCNT reinforcements along the grain boundaries.

It is evident from the SEM micrographs that the formation of hard magnesium precipitates enhances the hardness of the hybrid composite. SEM micrographs also depicted the absence of porosity, agglomeration, segregation, and blowholes, thereby enhancing the hardness of the hybrid composite compared to the Mg AZ91D matrix material.

### 3.3. Tensile Strength of AZ91D Hybrid Composites

**Figure 5:**
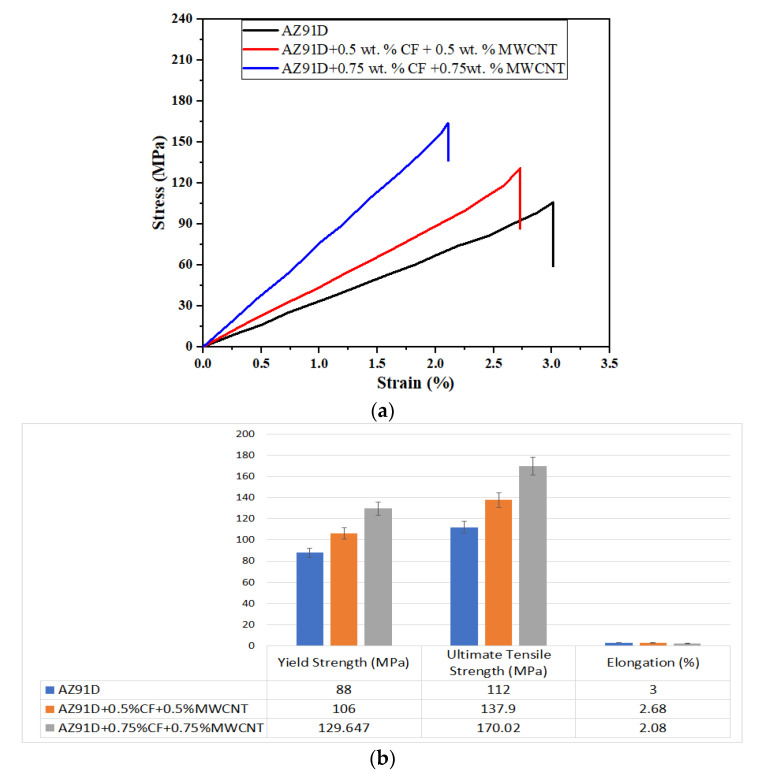
(**a**) Variation of yield strength, ultimate tensile strength, and elongation of Mg AZ91D hybrid composite with varying weight percentages of CF and MWCNT reinforcements; (**b**) stress–strain curves of all composites.

The stress–strain curves of all the metal-based composites are shown in Figure 5a. Figure 5b shows the variation of yield strength (YS), ultimate tensile strength (UTS), and percentage (%) of elongation of the Mg AZ91D hybrid composite reinforced with CFs and MWCNTs. As the percentage of the reinforcements increased, an increase in ultimate stress was observed, while a decrease in elongation was noted. Figure 5b revealed that the addition of CF and MWCNT reinforcements improved the yield strength and ultimate tensile strength of the hybrid AZ91D composite. The Mg AZ91D composite contains 0.75 wt.% CF and 0.75 wt.% MWCNT reinforcements, showing the highest UTS (23% and 51% higher) and YS (22% and 47% higher) compared to the Mg AZ91D composite containing 0.5 wt.% CF and 0.5 wt.% MWCNT, and Mg AZ91D, respectively. Similar behavior was also observed by the authors in the work on Mg composites [5,6]. Similarly, the percentage of elongation of the Mg AZ91D composite containing 0.75 wt.% CF and 0.75 wt.% MWCNT reinforcements show lower percentages (22% and 30% lower) compared to the Mg AZ91D composite containing 0.5 wt.% CF and 0.5 wt.% MWCNT, and Mg AZ91D.

The addition of hard CF and MWCNT reinforcements enhances the tensile strength of the hybrid Mg composite. As discussed earlier, the strong interfacial bond between the reinforcement and Mg AZ91D restricts the movement of dislocation and enhances the tensile strength of the Mg AZ91D hybrid composite. It has also been revealed by the microstructure that the uniform distribution of CF and MWCNT reinforcements improves the tensile strength of the Mg AZ91D hybrid composite. The increased tensile strength of the Mg AZ91D composite is also due to the improved hardness. The addition of CF and MWCNT reinforcements improves the hardness and tensile strength of the composite, thereby decreasing the elongation percentage of the composite.

**Figure 6:**
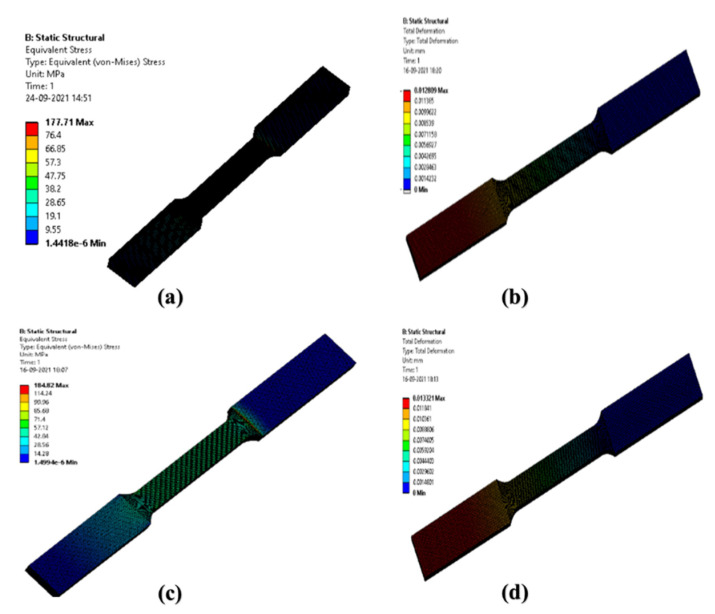
The analysis of the tensile strength of the AZ91D composite reinforced with CF and MWCNT reinforcements. (**a**,**b**): AZ91D + 0.5% CF’s + 0.5% MWCNT composite and (**c**,**d**): AZ91D + 0.75% CF’s + 0.75% MWCNT composite.

The static structural analysis is carried out using ANSYS 19.2 software. Hexahedron mesh was used for the analysis. The boundary conditions are applied with one end of the support fixed and a 2500 N force applied at another end. Equivalent von Mises stress is extracted and compared with a yield stress of experimental value. Equivalent stress and total deformation for specimen AZ91D + 0.5% CF’S + 0.5% MWCNT and specimen AZ91D + 0.75% CF’S + 0.75% MWCNT are 76.4Mpa and 114.24Mpa, and 0.01136 mm and 0.01184 mm, respectively. The results are shown in Figure 6. The error between experimental and simulation results is found to be less than 15% for AZ91D + 0.75% CF’S + 0.75% MWCNT, which is acceptable.

### 3.4. Impact Strength of AZ91D Hybrid Composites

The test is conducted in a universal Charpy testing machine as per the ASTM D256 standard. The energy absorbed by the specimen is determined, and thereby, the impact strength is evaluated. The dimensions of the specimen are 70 mm × 8 mm × 8 mm.

**Figure 7:**
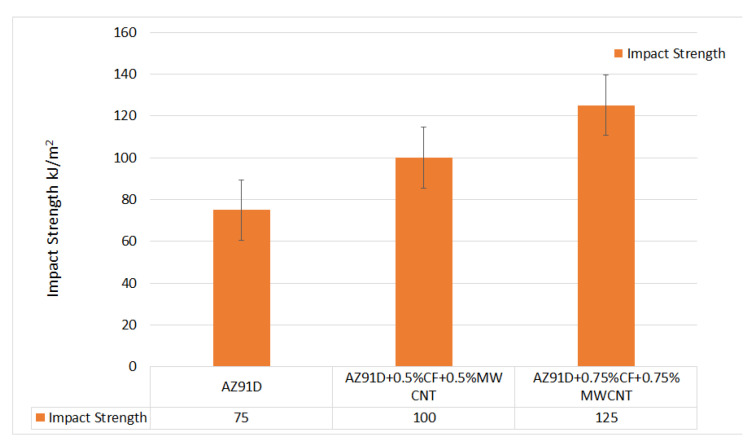
Variation of impact strength of Mg AZ91D hybrid composite with varying weight percentages of CF and MWCNT reinforcements.

From Figure 7, it is observed that the energy absorbed by the composite specimen and impact strength increases with the increase in the percentage of reinforcements. The bond between the elements becomes stronger as the percentage of reinforcement increases. The specimen AZ91d + 0.75% CF’s + 0.75% MWCNT has a higher impact strength of 125 kJ/m^2^. It is evidenced that, to a great extent, the incorporation of reinforcement particles into the magnesium matrix results in the improved impact strength of the base matrix.

### 3.5. Wear Rate of AZ91D Hybrid Composites

**Figure 8:**
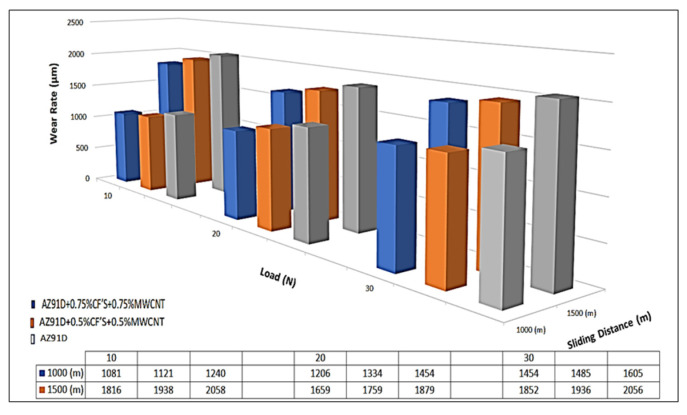
Wear rate of the Mg AZ91D hybrid composite with varying weight percentages of CF and MWCNT reinforcements.

A wear test is carried out on a pin in a disc tribo-tester to estimate the wear resistance of the Mg AZ91D hybrid composite with varying weight percentages of the CF and MWCNT reinforcements. Figure 8 shows the variation in the wear rate of the Mg AZ91D hybrid composite reinforced with CFs and MWCNTs. Figure 8 shows the higher wear for the Mg AZ91D compared to the Mg hybrid composite with varying CF and MWCNT reinforcements. The addition of CF and MWCNT reinforcements decreases the wear rate of the Mg AZ91D hybrid composite. The Mg AZ91D composite containing the 0.75 wt.% CF and 0.75 wt.% MWCNT reinforcements shows a lower wear rate, or higher wear resistance, compared to the Mg AZ91D composite containing 0.5 wt.% CF and 0.5 wt.% MWCNT, and Mg AZ91D. The coefficient of friction for the composite increases by increasing the wear rate [9,10,11]. The coefficient friction appears to be lower for the composite having a higher weight percentage of reinforcements.

As observed in the SEM micrographs, the improved wear resistance of the Mg AZ91D hybrid composite is due to the uniform distribution of CF and MWCNT reinforcements. The nano reinforcements act as a lubricating agent between the disc and the specimen, resisting the wear of the Mg AZ91D matrix material during sliding. The presence of magnesium precipitates influences the improved wear resistance of the Mg AZ91D hybrid composite. The improved hardness and tensile strength also enhance the wear resistance of the AZ91D composite [18,19].

### 3.6. SEM Micrographs of Worn Surfaces of AZ91D Hybrid Composites

**Figure 9:**
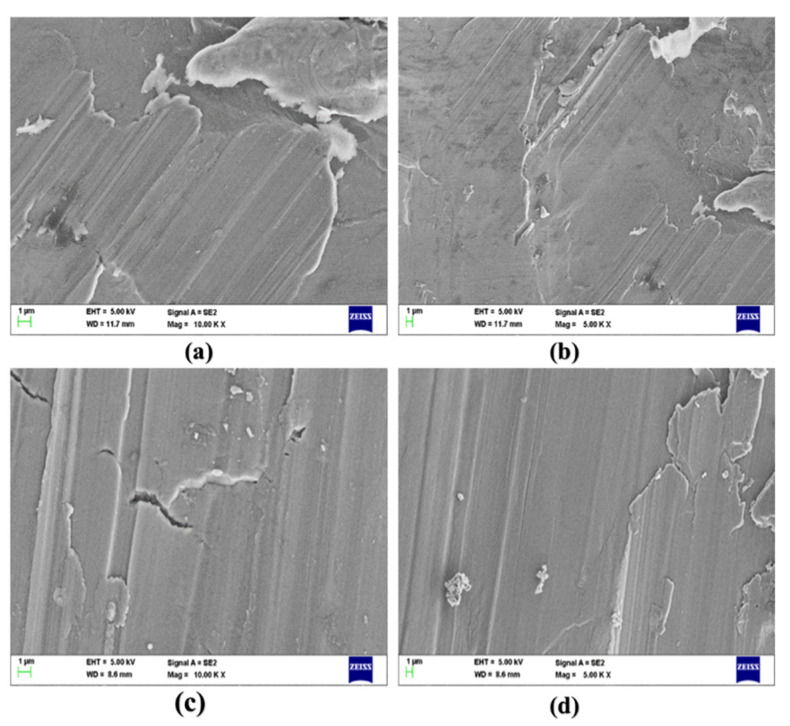
SEM micrographs of the worn surfaces of the Mg AZ91D hybrid composites (**a**) AZ91D + 0.5% CFs + 0.5% MWCNT composite with magnification 10.00 KX; (**b**) AZ91D + 0.5% CFs + 0.5% MWCNT composite with magnification 5.00 KX; (**c**) AZ91D + 0.75% CF’s + 0.75% MWCNT composite with magnification 10.00 KX; and (**d**) AZ91D + 0.75% CF’s + 0.75% MWCNT composite with magnification 5.00 KX.

The SEM micrographs of the worn surfaces of the Mg AZ91D composite with varying weight percentages of CF and MWCNT reinforcements were examined. The SEM micrographs of the AZ91D + 0.5% CF’s + 0.5% MWCNT composite and the AZ91D + 0.75% CF’s + 0.75% MWCNT composite were examined after the wear test was carried out at a load of 30N, a sliding distance of 1500m, and a velocity of 1.5 m/s. Figure 9 shows the SEM micrographs of the worn surfaces of the Mg AZ91D hybrid composite with varying weight percentages of CF and MWCNT reinforcements. Figure 9 depicts the clear and fine wear tracks, surface cracks, and delaminated surfaces on the wear track surfaces. The Mg AZ91D composite shows a lower wear rate at low load conditions of 10N. However, at high load conditions of 30N, the effect of CF and MWCNT reinforcements on wear resistance was not convincing, and an increased wear rate was revealed.

**Figure 10:**
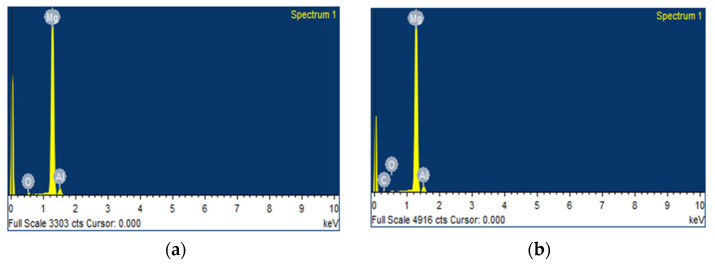
SEM-EDS analysis of the worn surfaces of the Mg AZ91D hybrid composite with varying weight percentages of CF and MWCNT reinforcements. (**a**): AZ91D + 0.5% CFs + 0.5% MWCNT composite; (**b**): AZ91D + 0.75% CFs + 0.75% MWCNT composite.

**Figure 11:**
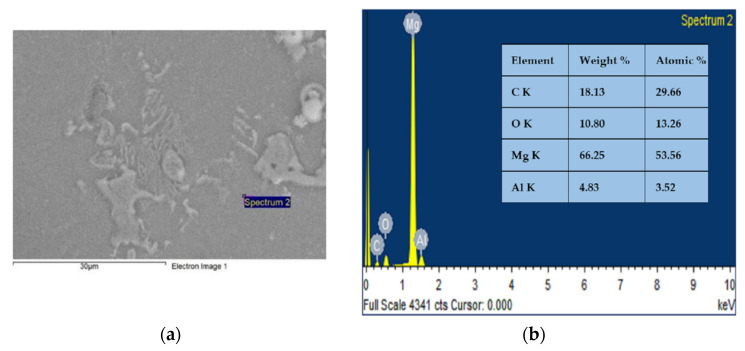
EDS analysis (**a**) depicts the presence of carbon (from CFs and CNTs) and oxygen, and (**b**) depicts other metals, such as Mg and Al.

Figure 10 shows the SEM-EDS analysis of the worn surfaces of the Mg AZ91D hybrid composite with varying weight percentages of CF and MWCNT reinforcements. It represents the formation of mechanically mixed layers during sliding wear. Due to the sliding wear at high load conditions of 30 N, the Mg AZ91D composite attains a substantial increase in temperature, which causes oxidation. The formation of oxide layers during sliding wear is evidenced in Figure 10, which shows the wear analysis of the AZ91D composite. The formation of oxide layers during sliding wear is confirmed and analyzed through the EDS of the worn surfaces. It is observed from the micrographs that delamination wear exists, and this is an indication of the presence of abrasive wear. The delamination occurs at the surface of the worn specimen due to the application of force, which causes the plastic deformation. Once the plastic deformation crosses the permissible limit, cracks initiate, delaminating the worn surfaces of the AZ91D composite, which separates from the specimen [20]. Figure 11 shows the presence of Mg, C, and O.

The uniform reinforcement could not be identified from the SEM images, as it is a small amount dispersed in the metal alloy. However, we have a clear EDS analysis picture that shows the presence of carbon (from CFs and CNTs) and oxygen, along with other metals, such as Mg and Al, as shown in Figure 11. We have mentioned the area spectra EDS analysis for a better understanding of the reaction.

## 4. Conclusions

The Mg AZ91D hybrid composite was successfully synthesized by reinforcing the CF and MWCNT reinforcements. The SEM micrographs showed the presence and homogeneous distribution of reinforcements along the grain boundaries of the cast Mg AZ91D composite. The mechanical and tribological tests were conducted as per ASTM standards. The test results showed that the hardness, tensile strength, and impact strength improved by 19%, 35%, and 66%, respectively. The mechanical and tribological properties of the composite improved due to the addition of hybrid reinforcements. Furthermore, the reinforcements also acted as lubricating agents and supported the base Mg AZ91D composite during sliding wear. The wear micrographs revealed the presence of abrasive wear. The EDS analysis of the wear micrographs showed the presence of Mg, carbon (C), and oxygen (O). The new class of magnesium metal matrix-based hybrid composites could be used in automotive and structural applications.

## Data Availability

Not applicable.
